# Chromosome instability region analysis and identification of the driver genes of the epithelial ovarian cancer cell lines A2780 and SKOV3

**DOI:** 10.1111/jcmm.17893

**Published:** 2023-07-31

**Authors:** Jianxiong Li, Zexin Chen, Wentao Xiao, Huaguo Liang, Yanan Liu, Wenqi Hao, Yongli Zhang, Fengxiang Wei

**Affiliations:** ^1^ Department of Gynecology Longgang District Maternity and Child Healthcare Hospital of Shenzhen City (Longgang Maternity and Child Institute of Shantou University Medical College) Shenzhen China; ^2^ Department of Cell Biology and Medical Genetics, School of Basic Medical Sciences Guangdong Pharmaceutical University Guangzhou China; ^3^ The Genetics Laboratory Longgang District Maternity and Child Healthcare Hospital of Shenzhen City (Longgang Maternity and Child Institute of Shantou University Medical College) Shenzhen China

**Keywords:** copy number variation, driver gene, serous ovarian cancer, single‐nucleotide variation, whole‐exome sequencing

## Abstract

Epithelial ovarian cancer (EOC) is one of the most prevalent gynaecological cancers worldwide. The molecular mechanisms of serous ovarian cancer (SOC) remain unclear and not well understood. SOC cases are primarily diagnosed at the late stage, resulting in a poor prognosis. Advances in molecular biology techniques allow us to obtain a better understanding of precise molecular mechanisms and to identify the chromosome instability region and key driver genes in the carcinogenesis and progression of SOC. Whole‐exome sequencing was performed on the normal ovarian cell line IOSE80 and the EOC cell lines SKOV3 and A2780. The single‐nucleotide variation burden, distribution, frequency and signature followed the known ovarian mutation profiles, without chromosomal bias. Recurrently mutated ovarian cancer driver genes, including LRP1B, KMT2A, ARID1A, KMT2C and ATRX were also found in two cell lines. The genome distribution of copy number alterations was found by copy number variation (CNV) analysis, including amplification of 17q12 and 4p16.1 and deletion of 10q23.33. The CNVs of MED1, GRB7 and MIEN1 located at 17q12 were found to be correlated with the overall survival of SOC patients (MED1: *p* = 0.028, GRB7: *p* = 0.0048, MIEN1: *p* = 0.0051), and the expression of the three driver genes in the ovarian cell line IOSE80 and EOC cell lines SKOV3 and A2780 was confirmed by western blot and cell immunohistochemistry.

## 1 INTRODUCTION

Ovarian cancer (OC) has a high morbidity and mortality rate and is a dangerous and fatal gynaecologic malignancy, with approximately 310,000 new cases and 200,000 deaths worldwide every year, while statistics from 2020 showed that there were approximately 60,000 cases of OC in China.^1^, ^2^ Epithelial ovarian cancer (EOC) accounts for approximately 90% of all ovarian malignancies, with 46% of women surviving 5 years after diagnosis contributing to the major factors of high mortality, usually with advanced disease at the time of diagnosis.^3^, ^4^ Currently, there are few specific diagnostic and treatment modalities for EOC. All women diagnosed with EOC should undergo germline or somatic breast cancer susceptibility gene (BRCA) genetic testing before proceeding to the upfront and recurrent setting.^5^, ^6^ The first‐line treatment for EOC mainly includes surgery and adjuvant chemotherapy.^7^ Treatment and management of OC can improve symptoms but limit long‐term survival after surgical resection.

EOC is a highly heterogeneous disease, with serous ovarian cancer (SOC), clear cell, endometrioid and mucinous ovarian cancer being common, with different characteristics of mutations in different tissue subtypes,^8^, ^9^ and histological subtypes may respond differently to treatment.^10^ Therefore, patients who benefit from targeted drugs can be identified by finding genomic biomarkers. Of these, high‐grade SOC is Type II EOC. In addition, Type II EOC initially exhibits significant chromosomal irregularities and becomes more unstable as the condition progresses, and advanced tumours account for a large proportion of these^8^; hence, the search for genomic variants of this type is very urgent. Copy number variation (CNV) is the major structural genomic variation involved in evolutionary adaptation, genomic disease and tumour progression.^11^ In many cancers, CNVs contribute to complex and heterogeneous genomic profiles. This makes it difficult to understand and uncover the unique molecular dynamics that shape the disease while preventing clinically effective patient typing. The analysis of CNV signatures represents a novel genomic stratification tool for probing this complexity, providing a sophisticated framework for the derivation of CNV patterns at the molecular level.^12^ This allows for the uncovering of the underlying genomic mechanisms of EOC, thereby potentially identifying therapeutic targets and prognostic correlates for SOC.

Along with advances in technology, several groundbreaking studies have shown the genetic background of EOC, including amplifications on chromosomes 19q13.42 (LILRA6), 3q26.2 (MECOM) and 8q24.3 (EEF1D) and deletions on chromosome 19q13.2 (CYP2A7).^13^, ^14^ Although several studies have revealed biomarkers of the molecular mechanisms of EOC progression and prognosis, a large part of the genetic mechanism associated with EOC development remains undiscovered.

As the basic material of the experiment, the genome of an OC cell line is relatively stable. In this study, whole‐exome sequencing was conducted on the EOC cell lines SKOV3 and A2780 to identify therapeutic targets and pathways. In somatic single‐nucleotide variation (SNV) mutations implicated in the progression of EOC, Gene Ontology (GO) and Kyoto Encyclopedia of Genes and Genomes (KEGG) analysis were used to accomplish pathway and function enrichment, and CNVs were examined. The connection between candidate genes and SOC patient survival was examined using The Cancer Genome Atlas (TCGA) patient data. The XIANTAO online platform was used for differential expression analysis of candidate targets, and cell validation experiments of three target driver genes, MED1, GRB7 and MIEN1, were conducted. The findings of this study advance knowledge of the molecular causes of EOC, as well as possible prognostic biomarkers and efficient treatment targets of SOC.

## 2 MATERIALS AND METHODS

### 2.1 Cell culture

Normal ovarian cells IOSE80 were cultured in RMPI‐1640 medium (Corning) containing 10% foetal bovine serum (Zhejiang Tianhang Biotechnology Co., Ltd.) and 1% Penicillin–Streptomycin (Anhui Biosharp Biotechnology Co., Ltd.), incubated in an incubator at 37°C with 5% CO_2_, and passed every 2–3 days. EOC cells SKOV3 were cultured in McCoy's 5A medium (Shanghai BasalMedia Biotechnology Co., Ltd.). EOC cells A2780 were cultured in the medium RPMI‐1640, with the addition of 2% glutamine (Wuhan Procell Life Science & Technology Co., Ltd.), and the other conditions were the same as above. IOSE80 and A2780 was derived from EXPASY. SKOV3 was derived from ATCC. All the cells were purchased from suyan biotech.

### 2.2 DNA extraction and quality assessment

1 × 10^5^ cells was added to the preheated 56°C 2 mL EP tube loading with 1 mL lysis buffer including 100 μL 20 mg/mL proteinase K and 100 μL 20% SDS and incubated at 56°C for 60–120 min. The tube was centrifuged at 18213 g for 10 min to get the supernatant after cooling to room temperature, adding an equal volume of phenol‐chloroform extraction and centrifuged at 18213 g for 10 min. Adding 2/3th volume of supernatant of isopropyl alcohol in 1.5 mL EP, then it was inverted at least three times and placed at −20°C for 2 h for precipitation. To remove the supernatant, the tube was centrifuged for 10 min getting the DNA pellet and it was washed with 1 mL 75% ethanol. Resuspend the pellet by centrifuging at room temperature and completely remove the supernatant. Air‐dry the DNA pellet in the biosafety cabinet for a few min and add 25–100 μL of TE buffer to dissolve the DNA pellet. DNA concentration was detected by Qubit Fluorometer (Thermo Fisher Scientific Co., Ltd.). Sample integrity and purity were detected by Agarose Gel Electrophoresis.

### 2.3 Library construction and whole‐exome sequencing

One microgram genomic DNA was randomly fragmented by Covaris. Fragmented DNA was selected by Agencourt AMPure XP‐Medium kit to an average size of 200–400 bp. The selected fragments were through end‐repair, 3′ adenylated, adapters‐ligation and PCR amplifying and the products were recovered by the AxyPrep Mag PCR clean up Kit. A certain amount of PCR products was taken for hybridisation with BGI Hybridization and Wash kits. After that, repeated PCR amplifying as before. The double stranded PCR products were heat denatured and circularized by the splint oligo sequence forming the single‐strand circle DNA (ssCir DNA) as the final library was qualified by quality control (QC). The library was amplified to make DNA nanoball (DNB) which have more than 300 copies of one molecular. The DNBs were loaded into the patterned nanoarray and pair‐end 100 bases reads were generated in the way of sequenced by combinatorial probe‐anchor synthesis on BGISEQ‐500 platform (BGI).

### 2.4 Alignment and somatic mutation calling

The original image data obtained by sequencing were converted into raw reads by DNBSEQ Base Calling, which was stored in FASTQ file format as raw data. Sequencing reads were aligned to the human reference genome (hg38) using BWA.^15^ In order to ensure the accuracy of variation detection, we conducted analysis according to the optimal variation detection analysis process recommended by GATK official website. GATK labelled repeated reads and base quality recalibration were used to compare the results. Based on the comparison results, the sequencing depth, coverage and comparison rate of each sample were statistically analysed. Duplicated reads marked by Picard were removed. Indel regions were realigned using GATK.^16^ Somatic mutations were called by MuTect2. All somatic variants were annotated in the single–nucleotide polymorphism database (dbSNP), the 1000 Genomes Project and EXAC by ANNOVAR. Analysis of mutation signature and visualisation using R package maftools.^17^

### 2.5 Copy number variation analysis in focal genomic regions

Paired reads were aligned to the hg38 reference genome using the BWA command and then sorted and indexed using SAMtools. CNVkit^18^ was used to analyse the CNV of somatic cells, and the log_2_ ratio was calculated using A2780 and SKOV3 cell lines as tumour samples and IOSE80 as normal reference samples. CNVkit algorithm was used to construct reference library with all samples, and then, the copy number of a single sample chromosome segment was calculated. GISTIC2.0^19^ was used to identify focal genomic regions with significant increases in CNA frequency.

### 2.6 GO and KEGG analysis

GO analysis is commonly used to annotate genes and their products, whereas the KEGG pathway database is used to identify functional and metabolic pathways for the mutated gene. We used the Database for Annotation, Visualization, and Integrated Discovery to perform GO and KEGG functional enrichment analyses for the mutated gene. GO enrichment results of biological process (BP), cellular component (CC) and molecular function (MF) were obtained using the R package. The KEGG pathway analysis of robust mutated gene was also conducted using the R package.

### 2.7 Bioinformatic analysis of clinical characteristics

The Xiantao tool (https://www.xiantao.love/products) was a comprehensive interactive web portal used to perform differential expression, survival and enrichment analysis in various cancer types. mRNA expression data and clinical information from TCGA database (https://cancergenome.nih.gov) and receiver operating characteristic (ROC) risk evaluation were analysed by the online tool XIANTAO platform. GEPIA2 (http://gepia.cancer‐pku.cn/) is a database that enables users to analyse RNA sequencing expression in a variety of ways, which was used to analyse target gene expression in different tumour stages. Clinical and survival data of SOC patients were collected from TCGA database and the correlation between candidate driver gene targets and survival of SOC patients was analysed according to CNV.

### 2.8 Western blot (WB) experiment

The protein expressions of GRB7, MED1 and MIEN1 in cell lysates of A2780, SKOV3 and IOSE80 were determined by WB. Proteins in total cell lysate were isolated by SDS‐PAGE. And then was transferred to the 0.45 μm PVDF membrane (Beyotime Biotechnology). After washing and blocking steps, the PVDF membrane was incubated with the following primary antibodies at 4°C overnight: anti‐GRB7 antibody (1:1000, #32977, Signalway Antibody), anti‐MED1antibody (1:1000, #DF6578, Affinity), anti‐MIEN1 antibody (1:1000, #DF7920, Affinity) and anti‐GAPDH antibody (1:1000, #GB11002, Servicebio). After washing the next day, the membrane was incubated with horseradish peroxidase bound secondary antibody. Bands were observed by an automated chemiluminescence image analysis system (Tanon‐5200) after the antibody was combined with enhanced chemiluminescence (Beyotime Biotechnology). Each sample was tested independently three times.

### 2.9 Cell immunohistochemistry (IHC) experiment

1 × 10^6^ cells were used to prepare A2780, SKOV3 and IOSE80 cell slides. GRB7, MED1 and MIEN1 staining were performed on triplet cell slides. The cell slide was dewaxed with xylene and rehydrated with a series of ethanol solutions and distilled water. Heat‐mediated antigen retrieval was performed in pH 9.0 Tris‐EDTA solution. The sections were closed and incubated with primary and secondary antibodies. The cell slides were developed by DAB and stained with haematoxylin. Cell immunohybridisation results under microscope (Olympus) observe. If there is yellow spot colouring in the background, it is judged as positive hybridisation result. If there is no obvious colouring, it is judged as negative hybridisation result. Each sample was tested independently three times.

### 2.10 Statistical analysis

IBM SPSS Statistics 26 was used for data analysis and R 4.1.3 was used for Kaplan–Meier survival analysis. *p* < 0.05 was considered statistically significant.

## 3 RESULTS

### 3.1 Landscape of somatic mutations in A2780 and SKOV3 cell lines

**FIGURE 1:**
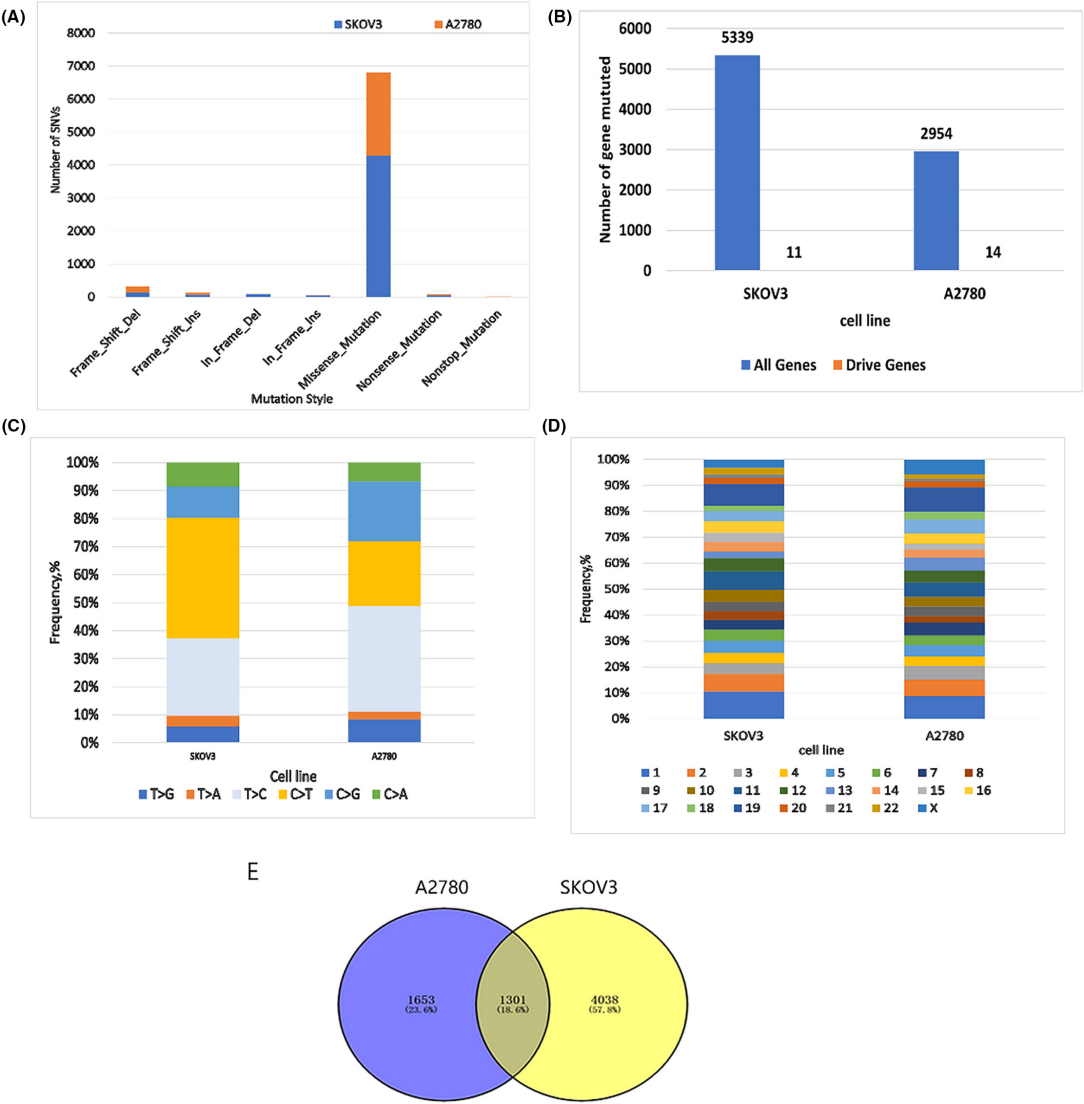
Landscape of single‐nucleotide variations (SNVs) in A2780 and SKOV3 cell lines. (A) The type and number of mutations in two cell lines. (B) The total number of mutated genes in the two cell lines and the ovarian cancer driver genes included in the Intogen database. (C) Type and proportion of base mutation in two cell lines. (D) The proportion of mutations in 23 pairs of chromosomes. (E) Venn diagram of the number of mutated genes in two cell lines.

The type distribution of SNVs shown in Figure 1A displayed that there were an average of 156.5 (145 and 168) frameshift deletion mutations, 64 (70 and 58) frameshift insertions, 47.5 (78 and 17) in frameshift deletions and 30 (47 and 13) in frameshift insertions in the A2780 and SKOV3 cell lines. There were 3402 (4297 and 2507) missense mutations, 41.5 (46 and 37) nonsense mutations, and 5.5 (9 and 2) nonstop mutations. A comparison with the Intogen database (www.intogen.org) of the somatic mutated genes involved revealed an average of 20 known OC driver genes (11 in SKOV3 and 14 in A2780) in the exons of the two cell lines (Figure 1B). The frequency of single‐nucleotide variation is shown in Figure 1C. Although the comparative analysis of mutation characteristics showed that the change patterns of different bases were generally similar, mainly T>G, C>T and T>C, the largest proportion of C>T was found in the SKOV3 cell line, while T>C was found in the A2780 cell line. Whether the difference between the two base changes points to different tumour origins or evolutionary patterns needs to be further explored. SNVs were also counted according to their chromosome distribution, and there was no evidence of chromosome bias between the two EOC cell lines (Figure 1D). In the two cell lines, SKOV3 involved more genes, with 4038 mutated genes, while the A2780 cell line had 1653 private mutated genes, and the two cell lines shared 1301 mutated genes (Figure 1E). The total SNV mutations in both cell lines are detailed in Figure S1.

### 3.2 Recurrent OC driver (IntOGen) in A2780 and SKOV3 cell lines and patterns of two signatures

**FIGURE 2:**
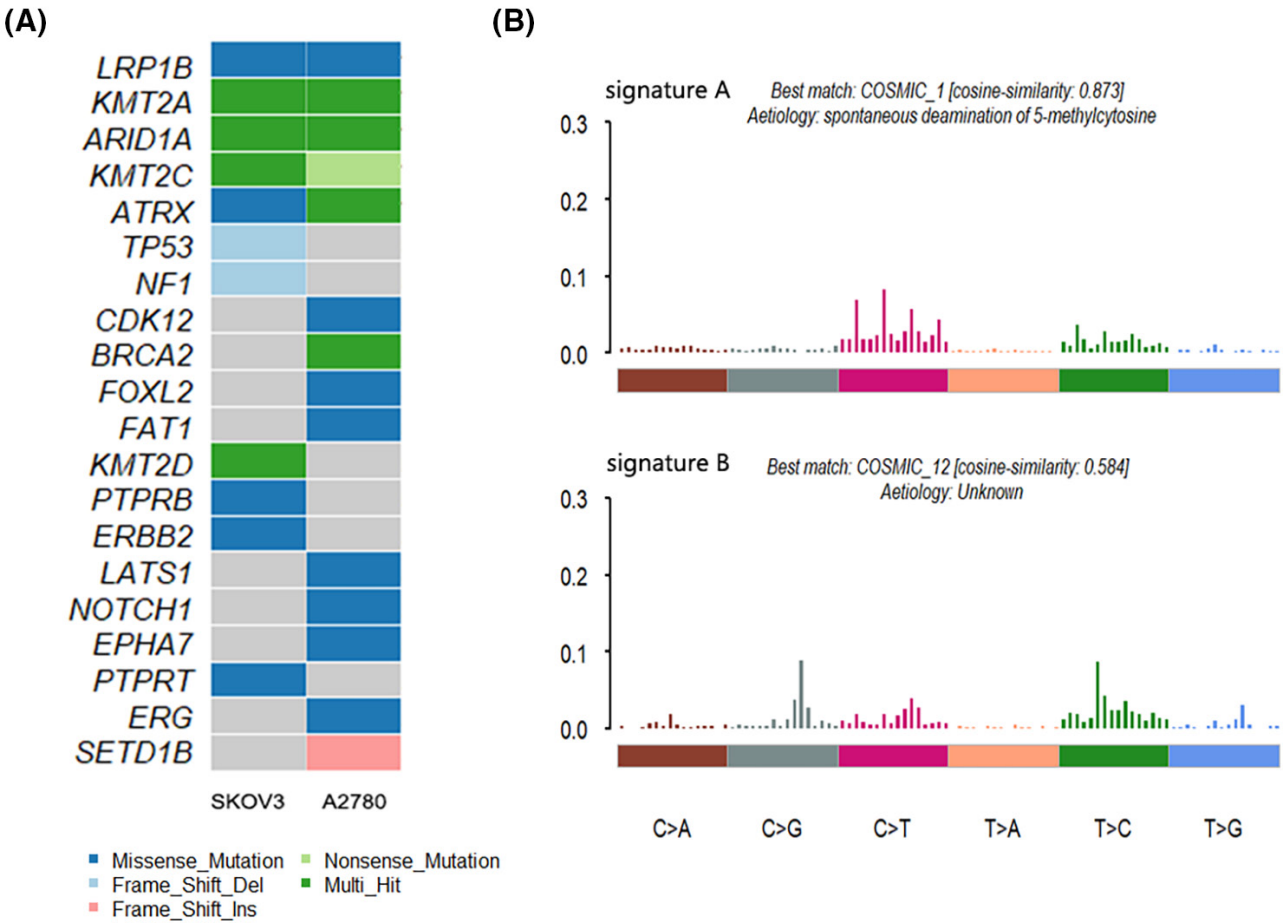
(A) Recurrent ovarian cancer driver in IntOGen found in epithelial ovarian cancer (EOC) cell lines. (B) Patterns of two signatures (Signatures A and B) identified in two EOC cell line genomes; the most similar COSMIC signature to each is also indicated.

In Figure 2A, multiple OC driver genes were identified as being shared by two EOC cell lines. Twenty OC driver genes were found in the IntOGen database from two cell lines. LRP1B, KMT2A, ARID1A, KMT2C and ATRX were mutated in both cell lines and the mutation sites caused caused changes in amino acid types as shown in Figure S2. ARID1A is the gene with the most SNV mutations. Changes in the expression of numerous genes (CDKN1A, SMAD3, MLH1 and PIK3IP1) caused by mutations in ARID1A contribute to the development of cancer and have been linked to the PI3K/AKT pathway in cell translocation,^20^ which was highly likely to become a therapeutic target for OC.^21^ TP53, a common tumour driver that is mutated in 96% of high‐grade SOC patients,^22^ was mutated only in SKOV3 cell lines and not in A2780 cell lines. In addition, these EOC cell lines also carry private driver gene mutations that have previously been reported in multiple cancer types, including OC. Mutation signature analysis of 96 substitution patterns identified two signatures in the two cell lines (signatures A and B, Figure 2B). Signature A was characterized by dominant C>T mutations and was highly similar to COSMIC signature 1 of C>T substitution at the NpCpG trinucleotide described previously (cosine correlation similarity = 0.873), which was identified as being related to spontaneous deamination of 5‐methylcytosine. Another mutation signature, signature B, was similar to the previously described COSMIC signature 12 (cosine correlation similarity = 0.584), which exhibits strong transcriptional chain bias and may reflect the involvement of transcription‐coupled nucleotide excision repair in large DNA adjuncts caused by exogenous carcinogens.

### 3.3 Mutated genes enriched by GO and KEGG pathway and function enrichment

**FIGURE 3:**
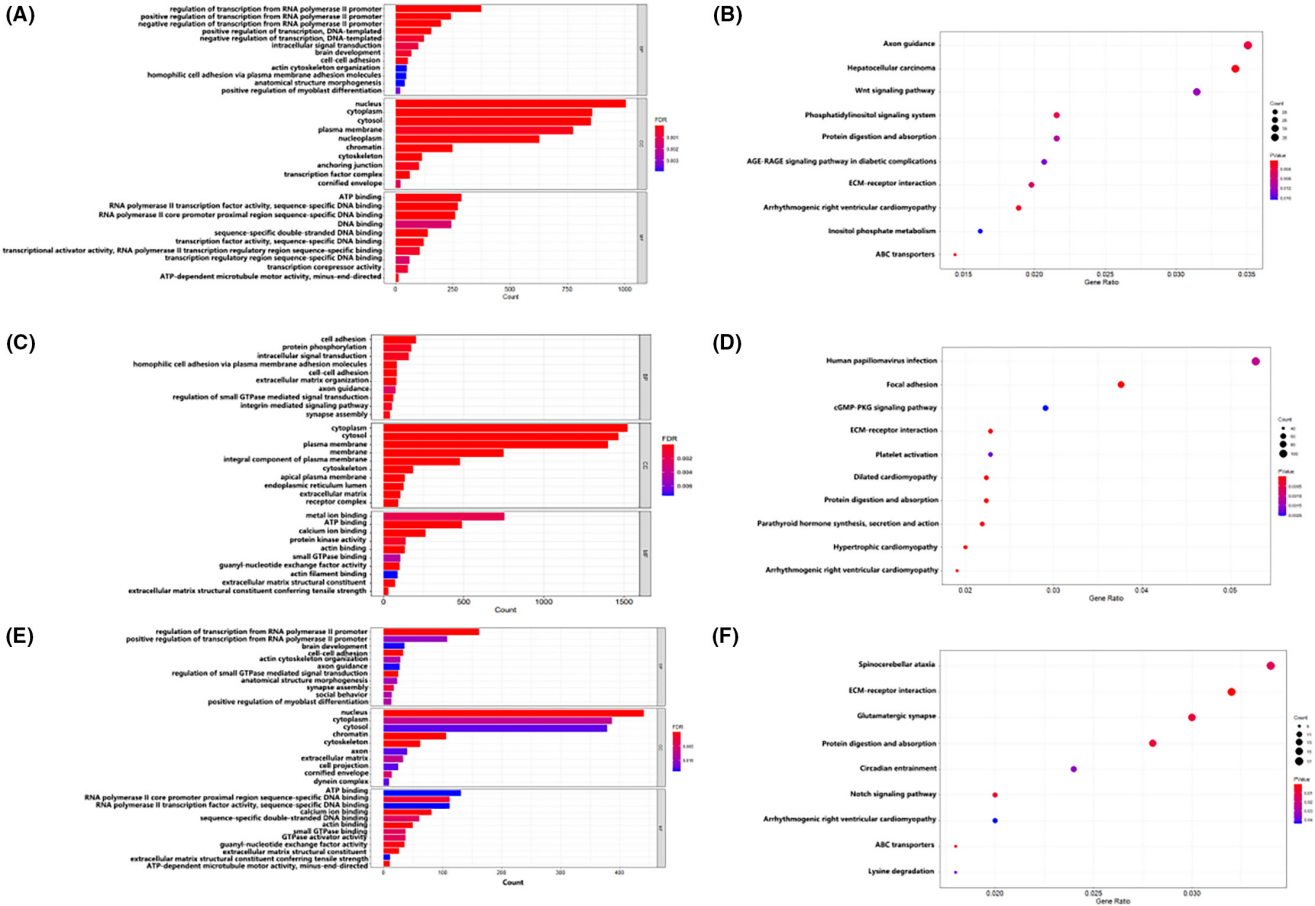
Gene Ontology (GO) and Kyoto Encyclopedia of Genes and Genomes (KEGG) pathway enrichment of mutation genes. (A) GO enrichment of A2780 mutation genes. (B) KEGG pathway enrichment of A2780 mutation genes. (C) GO enrichment of SKOV3 mutation genes. (D) KEGG pathway enrichment of SKOV3 mutation genes. (E) GO enrichment of common mutation genes. (F) KEGG pathway enrichment of common mutation genes.

We performed GO and KEGG analyses to evaluate the biological significance of mutated genes in EOC. Three functional domains were included in the GO analysis: CC, BP and MF. The top 10 in BP, CC and MF items that were enriched by A2780, SKOV3 and common mutant genes of two cell lines are shown in Figure 3A,C,E. The two most abundant BP functional groups were enriched by the common mutant gene in both cell lines (Figure 3E), which is not surprising in the transcriptional regulatory functions of RNA polymerase II promoters, as these genes are involved in transcription to perform specific functions. Among CC, ‘nucleus’ was the largest enriched category and in the MF categories, slightly more genes were enriched in ‘ATP binding’. As a result, the genes corresponding to these significant GO entries might be crucial for SOC. As shown in Figure 3B, SNV mutations in the A2780 cell line showed that the top 10 were mainly enriched in axon guidance and hepatocellular carcinoma, and in the SKOV3 cell line, they were mainly enriched in ECM‐receptor interaction and dilated cardiomyopathy (Figure 3D). In both cell lines, we found significantly KEGG‐defined pathways, including ECM‐receptor interaction, ABC transporters, Notch signalling pathway, etc. The ECM‐receptor interaction route has been linked to OC in existing studies,^23^ and many important genes also have an impact on the growth of the disease through this pathway.^24^, ^25^, ^26^ The Notch signalling pathway, which is also crucial for most tissue determination and development, is dysregulated in solid tumours and human malignancies.^27^, ^28^, ^29^ Variations in several of these pathway genes were also discovered by integrated and epigenomic analyses of OC cell lines.^30^ This indicates that our above analysis results are credible.

### 3.4 CNV mutation landscape in A2780 and SKOV3 cell lines

**FIGURE 4:**
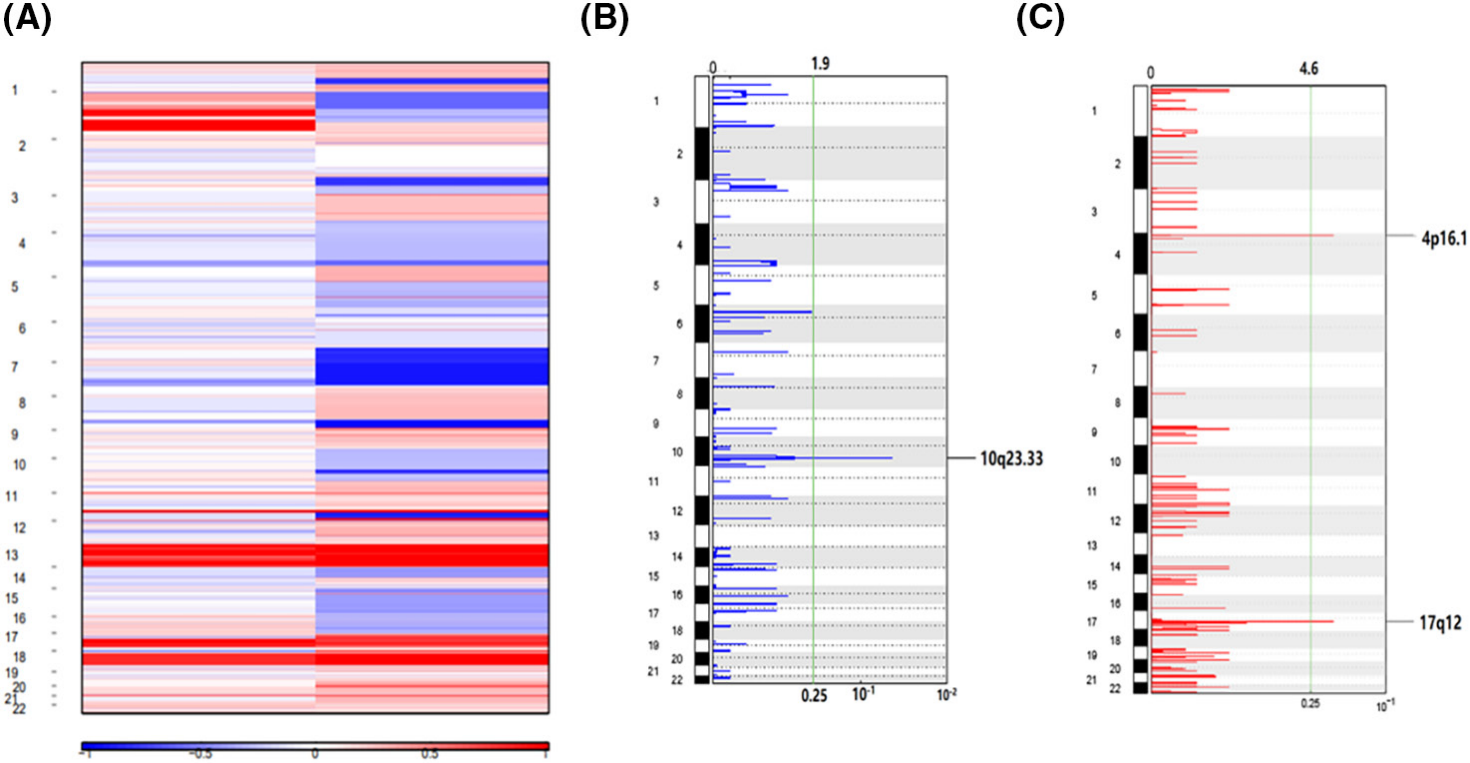
GISTIC analysis revealed the genome distribution of copy number alterations. (A) CNVs mutation landscape of each chromosome of two cell lines. (B) GISTIC *q*‐values (*x*‐axis) for deletions (blue) are plotted across the genome (*y*‐axis). (C) GISTIC *q*‐values (*x*‐axis) for amplifications (red) are plotted across the genome (*y*‐axis).

To identify more genes associated with serous EOC, we next evaluated a wider range of CNVs in A2780 and SKOV3 cells. There were still great differences in the variation in chromosome copy number between the two cell lines, as the IOSE80 normal ovarian epithelial cell line genome was used as reference (Figure 4A; Figure S3). Three focal genomic areas with significantly higher CNA frequencies were found in A2780 and SKOV3 cells using GISTIC2.0, one of which was a deletion (chromosome 10q23.33) and two of which were amplifications (chromosomes 4p16.1 and 17q12) (Figure 4B,C). Gains of 17q12 were specific for late‐stage OCs by array CGH analysis.^31^ This demonstrated that the patient's genetic makeup is shared by the OC cell line. It has been established that the CNV in the 10q23.33 region containing the oncogene PTEN is connected to various cancers, including colorectal cancer^32^ and gastric cancer.^33^ In the two cell lines, there were few CNVs. When TCGA SOC data were analysed, CNV amplification was shown in numerous places, and several of these sites contained oncogenes, including CCNE1 (19q12), MYC (8q24.21), PVT1 (8q24.21) and EPPK1 (8q24.3). Numerous genomic areas, including chromosome 6q (6q27 with AFDN and 6q25.1 with GRM1), showed a high frequency of deletions (Figure S4A,B). Although there were discrepancies in the results between the cell line and the patient, the patient's situation was highly heterogeneous, and there were some similarities between the results of the study of the cell line and the patient.

### 3.5 Overall survival analysis of candidate genes on SOC clinical patients

**FIGURE 5:**
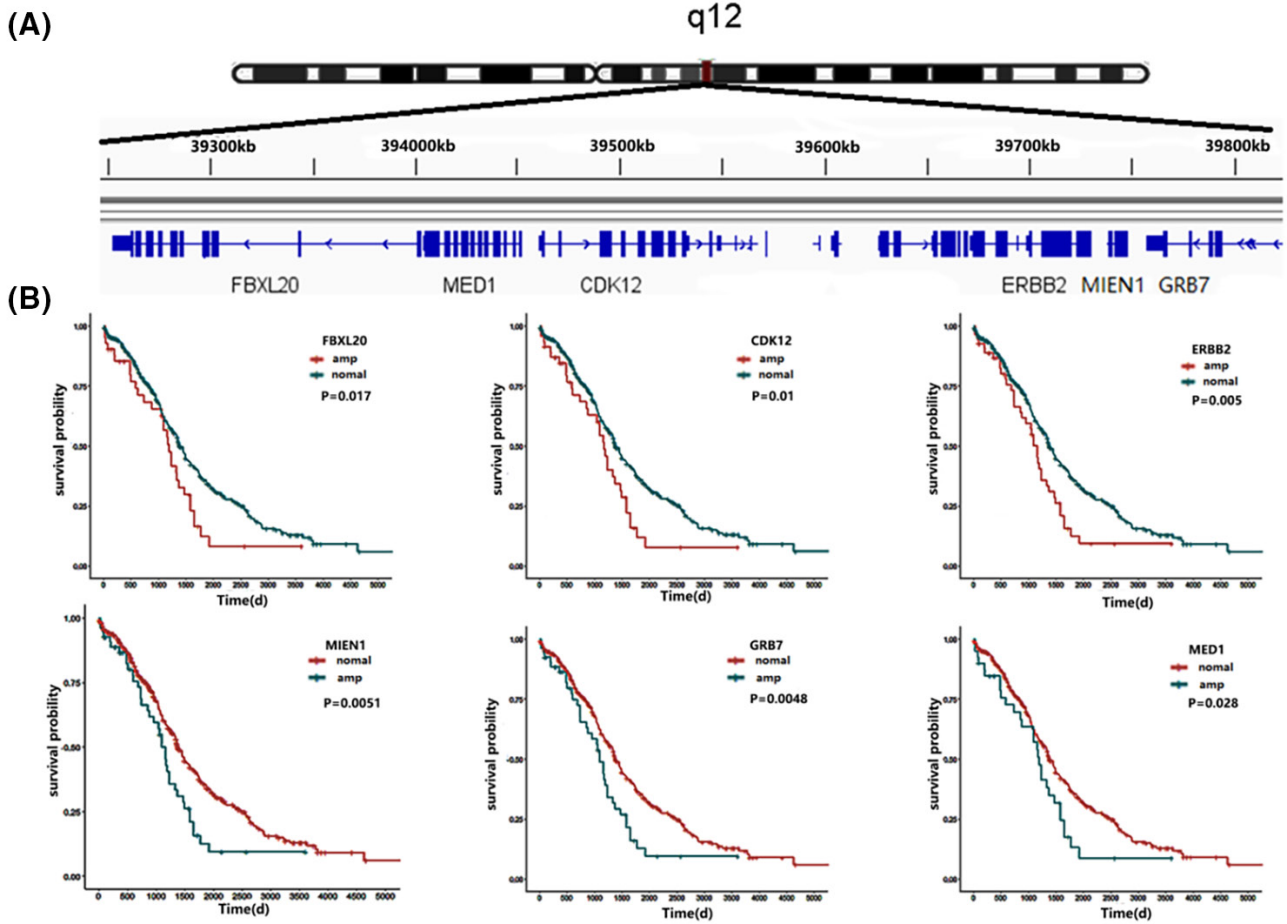
Overall survival analysis of six candidate genes (FXBL20, CDK12, ERBB2, MED1, GRB7 and MIEN1) were associated with serous ovarian cancer patients in chromosome 17q12. (A) The 17q12 genomic region includes six candidate genes significantly associated. (B) Survival curves of six candidate genes.

The TCGA database's association between overall survival (OS) for clinical patients and SOC was examined using Kaplan–Meier (K–M) survival analysis. In the region of chromosome 17q12, six candidate genes—FBXL20, CDK12, ERBB2, MIEN1, GRB7 and MED1—were removed; their chromosomal locations are depicted in Figure 5A. The copy number amplification of the candidate genes in this chromosome segment was discovered to be linked with the patients' OS by K–M survival analysis (*p* < 0.05, Figure 5B). Among them, the correlation of MIEN1, GRB7 and MED1 was particularly noteworthy. SOC patients' prognoses are impacted by the amplification of these genes, and the chances of survival are decreased by candidate gene mutations.

### 3.6 Expression of candidate genes in SOC clinical patients

**FIGURE 6:**
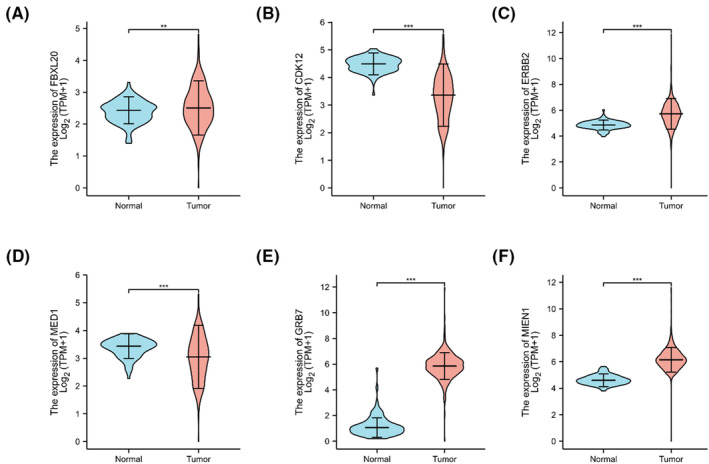
Gene expression of 6 candidate genes based on The Cancer Genome Atlas and GTEx databases. **p < 0.01, ***p < 0.001. (A) FXBL20. (B) CDK12. (C) ERBB2. (D) MED1. (E) GRB7. (F) MIEN1.

Regarding gene mRNA expression based on TCGA and GTEx databases of SOC clinical patients. Figure 6 illustrates how the expression of FBXL20, CDK12, ERBB2, MED1, GRB7 and MIEN1 varies between normal tissue and malignant tissue. While the expression levels of GRB7, MIEN1, ERBB2 and FBXL20 were considerably greater in tumour tissues than in normal tissues (*p* < 0.001 or *p* < 0.01), the expression levels of MED1 and CDK12 were significantly lower in tumour tissues than in normal tissues (*p* < 0.001). This suggested that the variance of these potential genes could lead to dysregulation of gene expression, which would therefore have an impact on SOC patients' quality of life. As a result, the focused drivers can be employed as molecular markers to aid in the clinical application and in the diagnosis, treatment, and prognosis of SOC.

### 3.7 Gene expression of driver genes with WB and cell IHC in vitro

**FIGURE 7:**
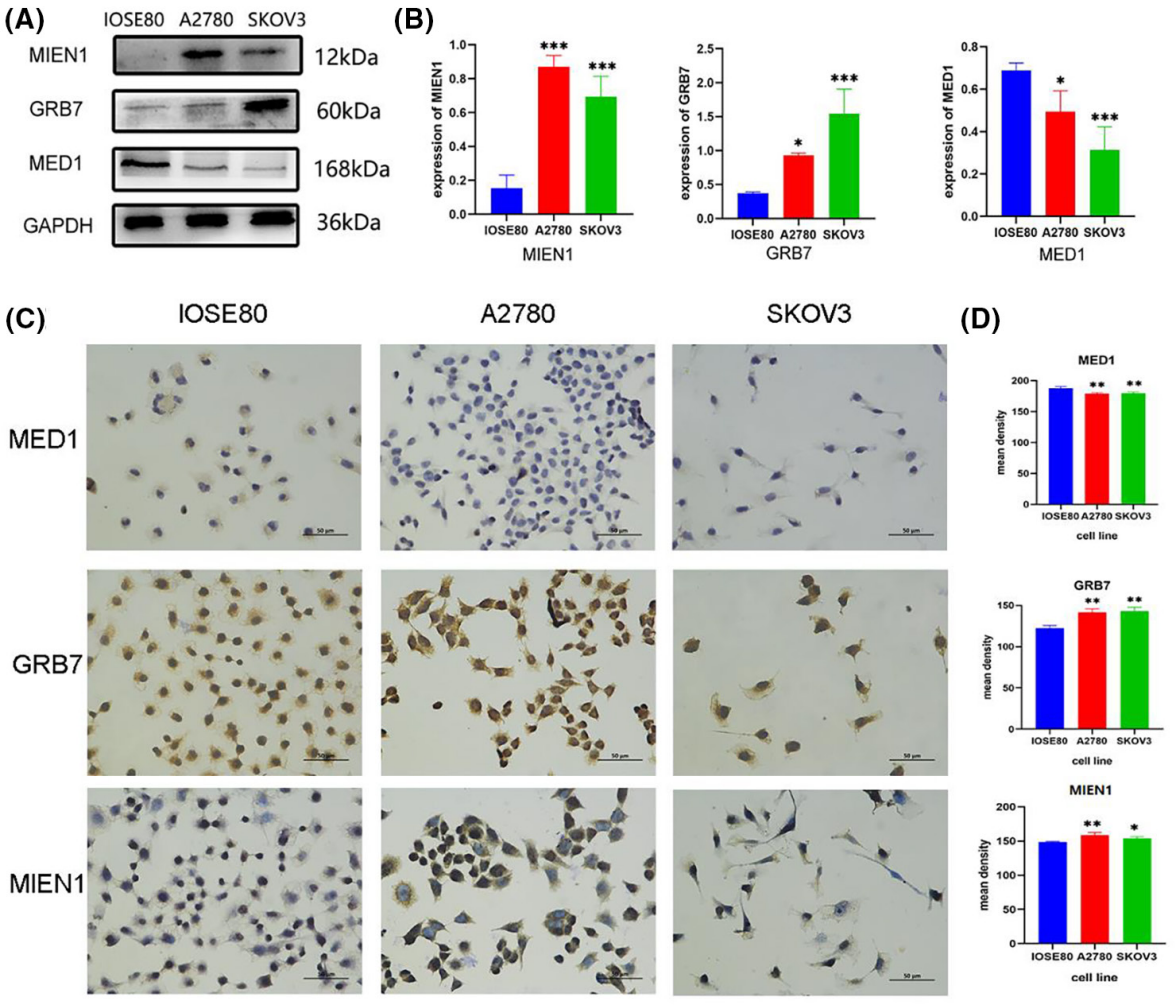
Gene expression of 3 driver genes (MIEN1, GRB7 and MED1) in 3 cell lines (IOSE80, A2780 and SKOV3). (A) Western blot results. (B) Statistical diagram of western blot results. (C) Cell immunohistochemistry (IHC) results. (D) Statistical diagram of cell IHC results. All experiments were conducted in triplicate. Data are expressed as mean ± standard deviation. *p < 0.05, **p < 0.01 and ***p < 0.001 compared with IOSE80 group.

OC incidence and progression are strongly correlated with MIEN1, GRB7 and MED1.^34^, ^35^, ^36^ These three genes are regarded as the focused driver genes of SOC based on the correlation results of genomic alterations in EOC cells and SOC clinical survival analyses. WB was used to examine the differences in MIEN1, GRB7 and MED1 expression between the normal ovarian cell lines IOSE80 and EOC cell lines to confirm the expression of the target gene in the mutant. MIEN1 and GRB7 expression were shown to be higher in SKOV3 and A2780 cell lines compared to the normal ovarian cell lines, and expression of MED1 decreased (Figure 7A,B). Cell IHC experiment yielded identical results (Figure 7C,D), which were in agreement with the TGCA and GTEx database analyses. The mutual validation of WB, IHC in vitro, and bioinformatics analysis results suggests that the analysis results of SOC driver genes were consistent and dependable.

## 4 DISCUSSION

Through whole‐exome sequencing reports of two commonly used EOC cell lines A2780 and SKOV3, we revealed the mutant landscape of EOC in this study. We also strongly support the use of these cell lines as experimental models in vitro to investigate the mechanisms that drive EOC origin and progression. Furthermore, a thorough comprehension of the mutations and new driver genes that activate and/or disrupt numerous signalling networks in these cell lines will aid researchers in creating experiments to test novel therapeutic approaches in suitable signalling environments and offer recommendations for the clinical use of molecular targets.

Both these cell lines and EOCs frequently have mutations (Figure 2A), such as NF1 and TP53, which has been linked to an increased risk of OC.^37^ The discovery of other, less prevalent mutations, such as PTPRT, but potentially significant cancer‐causing mutations has substantial ramifications for understanding past and upcoming research employing these cell lines. Both cell lines featured the same dysregulation of the Notch signalling pathway and the ECM‐receptor interaction pathway, according to KEGG analysis of the shared mutant genes (Figure 3F). Many academics also thought that one of the metastatic mechanisms for EOC may include the ECM‐receptor interaction.^23^, ^38^, ^39^, ^40^ The Notch signalling pathway, plays a crucial physiological role in the ovaries by controlling cell invasion, adhesion, proliferation, apoptosis and differentiation through cell contacts, and has been found to share a tight connection with OC.^41^ This is extremely important for the treatment of EOC in the future.

Multiple studies have already shown CNV signatures in predicting patient outcomes in both single tumours and multiple cancers.^42^, ^43^, ^44^ CNV plays a significant role in the pathophysiology of different malignancies, and the discovery of new cancer driver genes, especially in rare or understudied tumour cohorts, may be of further application using CNV signatures.^45^ From the analysis of TCGA database, there are many CNVs in SOC patients. We discovered amplification of 4p16.1 and 17q12 and deletion of chromosome 10q23.33 (Figure 4) in both cell lines. Although the CNV of the cell lines was not as large as that of the patient, the results showed that it was representative and corresponding. The findings of this study demonstrated that FBXL20, CDK12, ERBB2, MED1, GRB7 and MIEN1 in 17q12 were correlated with the OS of SOC (Figure 5), indicating that the change in the related gene's copy number was correlated with the prognosis of SOC patients. These genes were therefore considered to be potential candidate genes for SOC. According to earlier studies, FBXL20 gene amplification is linked to poor OS in OC patients,^46^ and compared to normal tissues, the patient's tissues exhibit elevated expression.^47^ The same results were found through our analysis of SOC (Figure 6A). OC is greatly impacted by the overexpression of FBXL20 because it encourages the growth of cells, prevents cell apoptosis and speeds up the cell cycle. In terms of ERBB2 amplification, several malignancies exhibit it.^48^, ^49^, ^50^ Both breast cancer and stomach cancer have received complete confirmation of the carcinogenic effect of ERBB2 amplification.^51^ According to research, the most frequently coamplified gene with ERBB2 in malignancies with a high ERBB2 amplification frequency is CDK12,^52^ and we also screened amplification of these two candidate genes in SOC tumours in our study. GRB7, MED1 and MIEN1 were the three most intriguing genes. The AUC values of GRB7 and MIEN1 were 0.992 and 0.983 (Figure S5), respectively, according to ROC analysis, demonstrating good diagnostic performance. The AUC value of MED1 was 0.654 between 0.5 and 0.7 which can also meet the diagnostic criteria, and the MED1 gene was closely associated with the stage of SOC.

**FIGURE 8:**
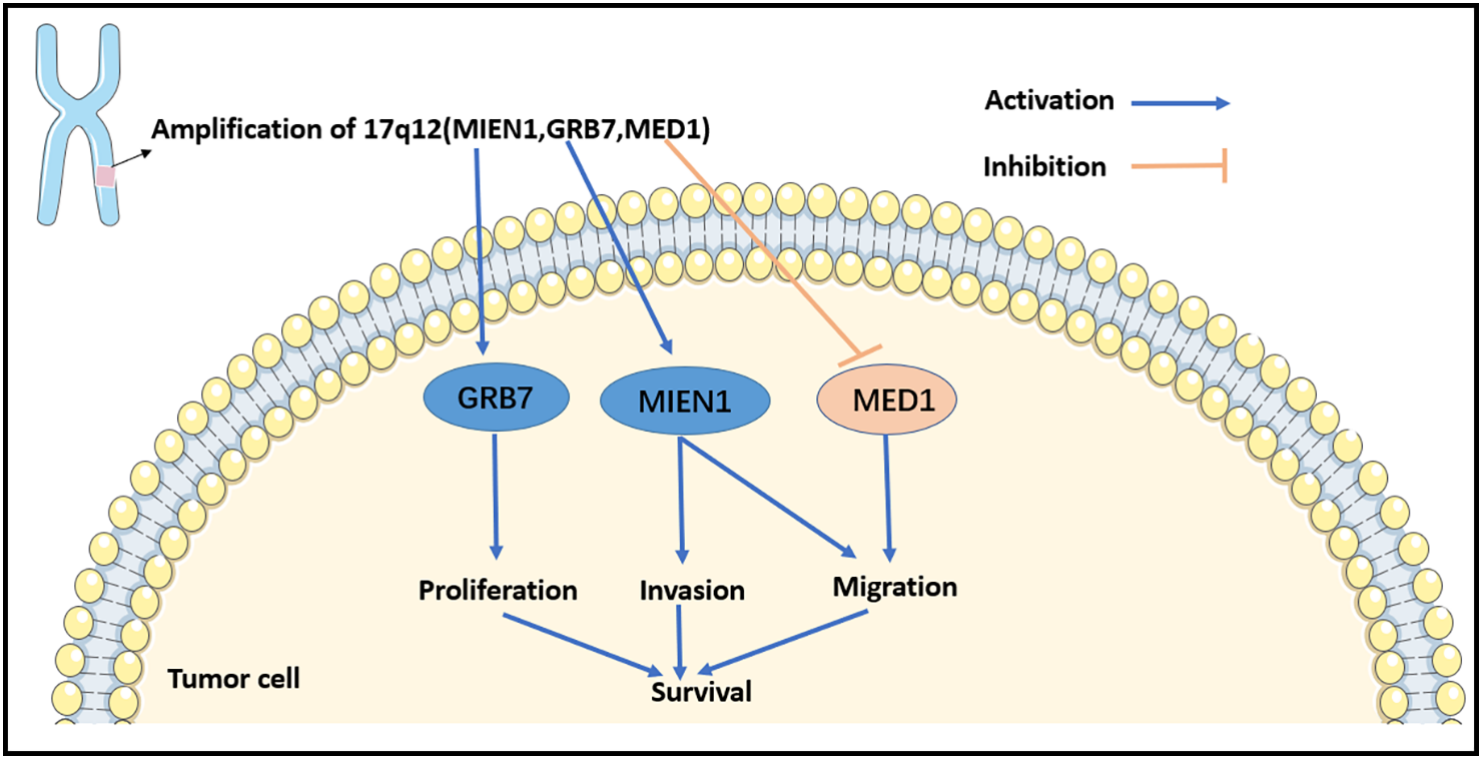
Effect of MIEN1, GRB7 and MED1 on survival of serous ovarian cancer.

MED1 is part of a highly evolutionarily conserved multiprotein mediator complex involved in the activation of gene transcription. Through biogenic K–M analysis, we discovered that MED1 copy number amplification was related to SOC patients OS, and WB and IHC experiments on two mutant cell lines also showed different expression levels of related proteins (Figures 5 and 7). Additionally, by analysing TCGA and GTEx data for MED1 in a large SOC cohort, we found significantly decreased MED1 expression between people without cancer and tumour patients (Figure 6D). Based on 427 SOC samples and 88 normal ovarian tissues, MED1 expression was negatively correlated with the clinicopathological staging of OC and the malignancy of SOC (Figure S6). These findings imply that the expression of MED1 may be downregulated as a marker for SOC patient development and prognosis. Low expression of MED1, has also been linked to another tumour type bladder cancer, and is associated with lymph node metastasis.^53^ The following phase involves performing additional analysis using biological studies with various cell lines and clinical samples. GRB7, a functional multidomain convergence protein that interacts with the activated epidermal growth factor receptor, has been demonstrated to be a critical regulator of numerous physiological and pathological processes through a variety of signalling pathways.^54^ This gene was significantly expressed in both of the mutant cell lines used in our investigation. Additionally, it has been noted that GRB7 has aberrant expression in several malignancies, including oesophageal cancer and bladder cancer, and they all confirmed that GRB7 knockdown can prevent the proliferation of cancer cells that are associated with it.^55^, ^56^ Our experimental results on EOC cells confirm and support the above reports (Figure 7). MIEN1 is a membrane‐anchored protein that has an important influence on the migration and invasion of cancer cells,^57^, ^58^ and higher MIEN1 levels are more related to cisplatin drug resistance in OC.^35^ Therefore, it is hypothesized that OC patients receiving chemotherapy who have high expression of MIEN1 may have a poor prognosis. Among all the genes we identified as potential SOC biomarkers, MIEN1 was unique and has not been reported in previous studies. On the one hand, we confirmed the upregulated expression result with cell experiments in vitro (Figure 7A–D) and predicted that MIEN1 has good diagnostic value in SOC (AUC = 0.983, Figure S5). Therefore, MIEN1 could serve as a novel biomarker of SOC. It can be seen from many studies above that MED1, GRB7 and MIEN1 are closely related to the proliferation, migration and invasion of tumour cells, which further affects SOC patient survival (Figure 8), and the three screening targets will be extremely important for the clinical application of SOC diagnosis, therapy and prognosis evaluation.

In conclusion, the current results are from bioinformatic analysis and cellular assays, and the three targets will then undergo functional confirmation at the cellular and histopathological levels. We identified some EOC somatic mutation driver genes by whole‐exome sequencing and bioinformatics analysis and on CNV analysis in the unstable chromosomal region 17q12 and found that amplification of MED1, MIEN1 and GRB7 correlated with SOC patient prognosis. The mutants were also differentially expressed and could therefore be used as potential targets for the treatment and diagnosis of SOC. To confirm their worth in SOC diagnostics, therapy and prognosis, this study provides the framework and direction for additional clinical trials that are also being considered.

## AUTHOR CONTRIBUTIONS

**Jianxiong Li:** Funding acquisition (equal); project administration (equal). **Zexin Chen:** Methodology (equal); visualization (equal). **Wentao Xiao:** Software (equal); writing – review and editing (equal). **Huaguo Liang:** Formal analysis (equal); methodology (equal). **Yanan Liu:** Writing – review and editing (equal). **Wenqi Hao:** Software (equal). **Yongli Zhang:** Methodology (equal); project administration (equal). **Fengxiang Wei:** Funding acquisition (equal); project administration (equal).

## FUNDING INFORMATION

This work was supported by the Medical and Health Technology Project of Shenzhen Longgang District (No. LGKCYLWS2021000023), the National Natural Science Foundation of China (No. 81102753), and the ‘ovarian cancer chromosome instability region molecular marker target and clinical application research’ enterprise horizontal project.

## CONFLICT OF INTEREST STATEMENT

The authors have no conflict of interest.

## SEQUENCE DATA

These sequence data have been submitted to the GenBank databases under accession number PRJNA994694.

## Supporting information


Figure S1.
Figure S2.
Figure S3.
Figure S4.
Figure S5.
Figure S6.

## Data Availability

The data sets generated during and/or analysis during the current study are available from the corresponding author on reasonable request.
