# Supplementary material for: Chromosome instability region analysis and identification of the driver genes of the epithelial ovarian cancer cell lines A2780 and SKOV3
Source: J Cell Mol Med. 2023 Jul 31;27(21):3259–70. doi: 10.1111/jcmm.17893 (PMC10623538; doi:10.1111/jcmm.17893)
Supplement: Supplementary file 1 — Figure S1. Figure S2. Figure S3. Figure S4. Figure S5. Figure S6. [file JCMM-27-3259-s001.pdf]

Supplementary Information

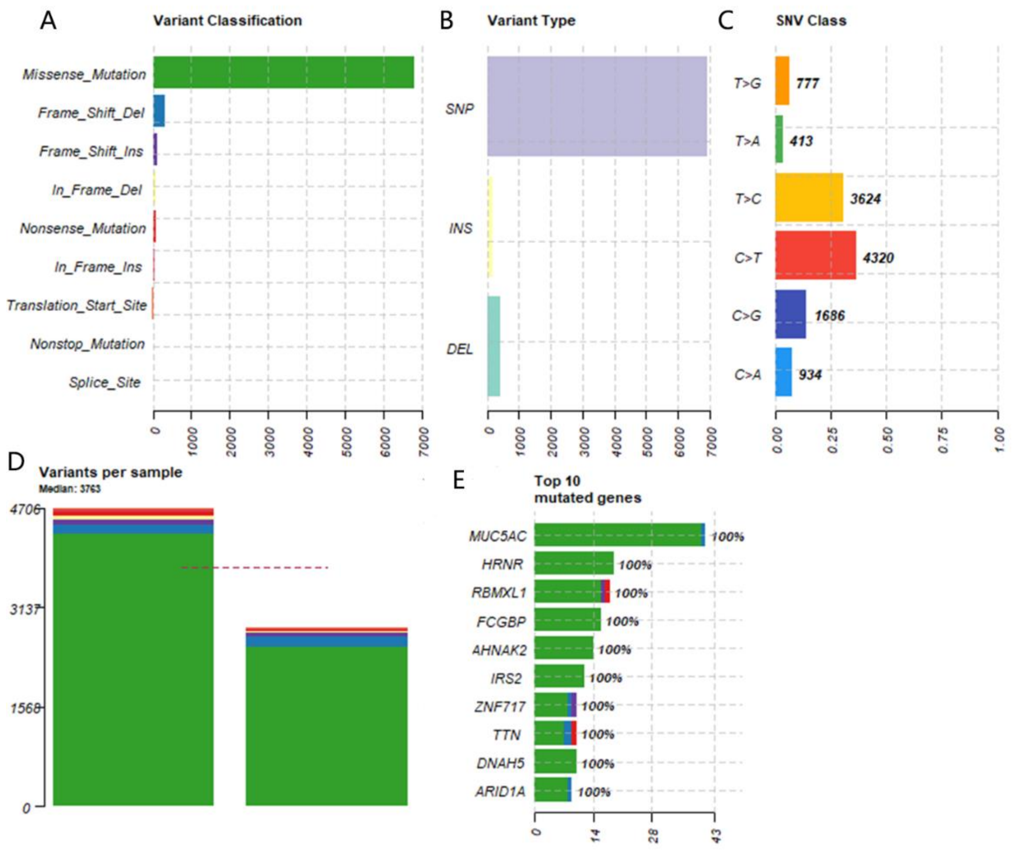

Figure S1 Landscape of SNVs in A2780 and SKOV3 cell lines. A. Total variant classification in both cell lines. B. Total variant type in both cell lines. C. Total SNV class in both cell lines. D. Variants in two cell lines. E. Top 10 mutated gene.

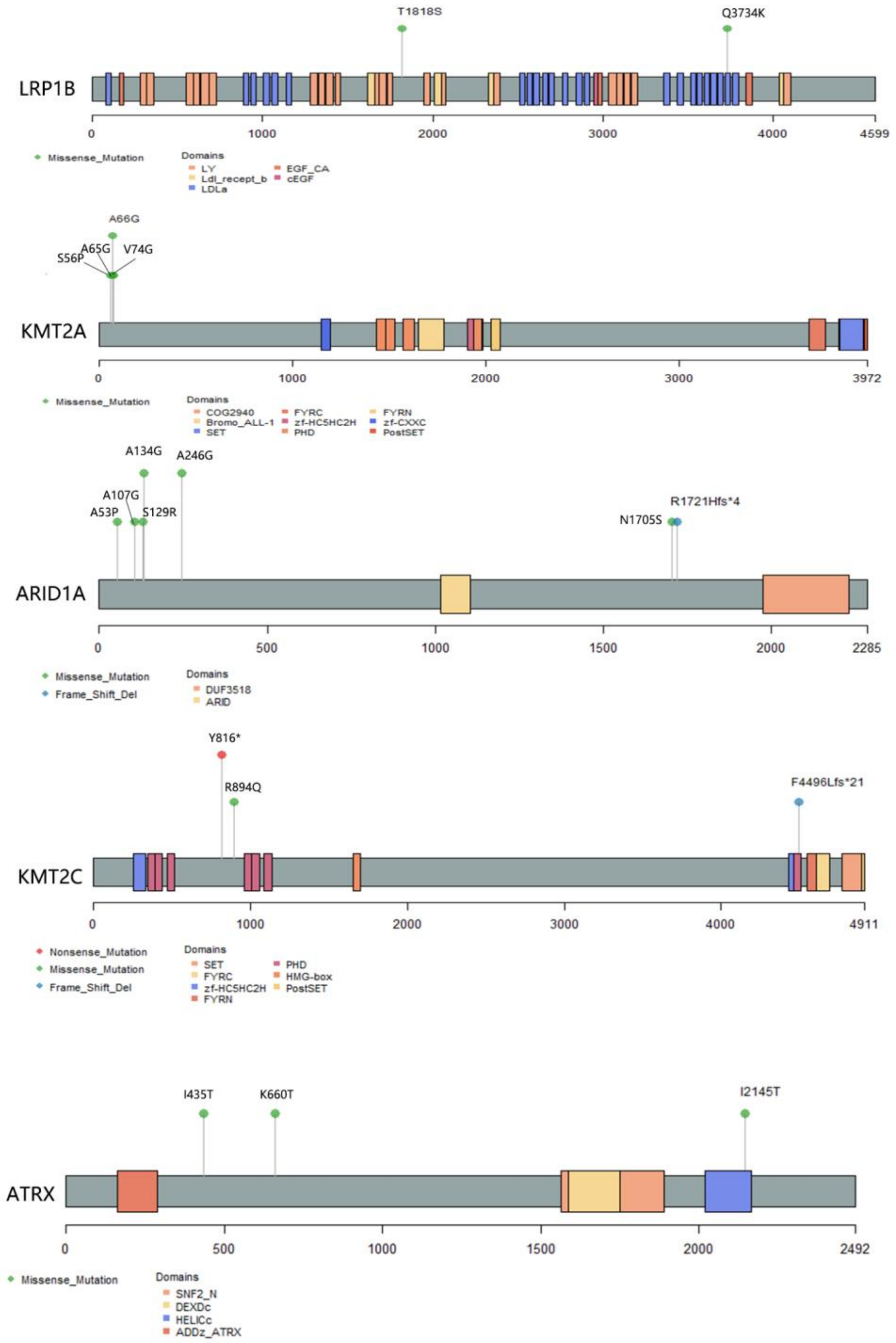

Figure S2 Schematic representation of SNVs and INDELs in common mutated genes LRP1B, KMT2A, ARID1A, KMT2C and ATRX.

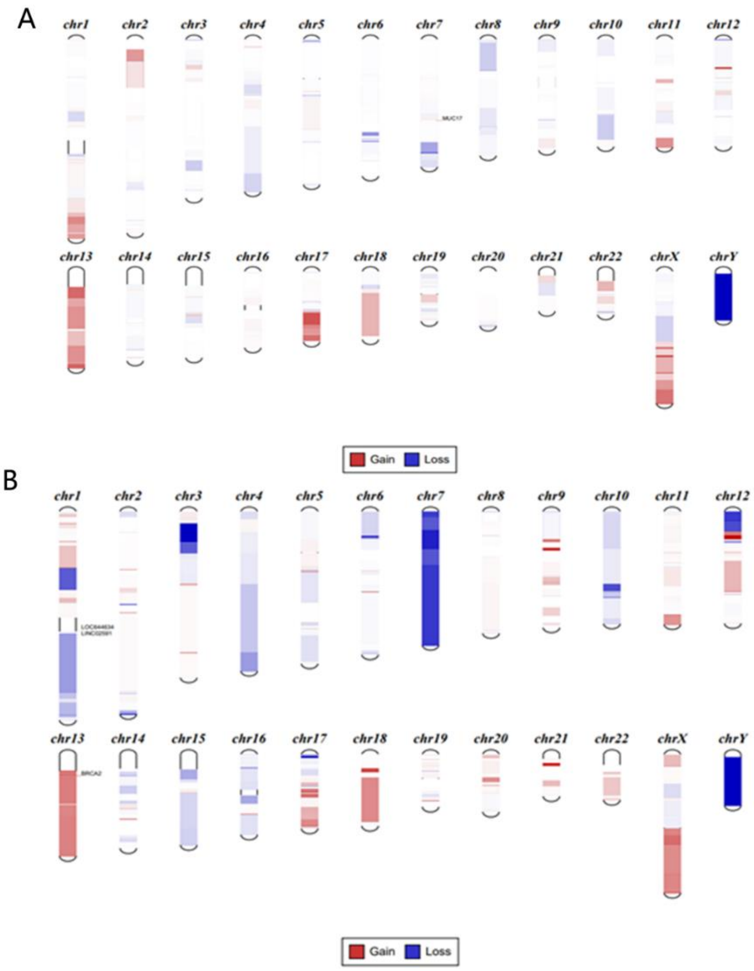

Figure S3 CNVs mutation landscape of each chromosome of two cell lines. A. A2780 copy number alterations. B. SKOV3 copy number alterations.

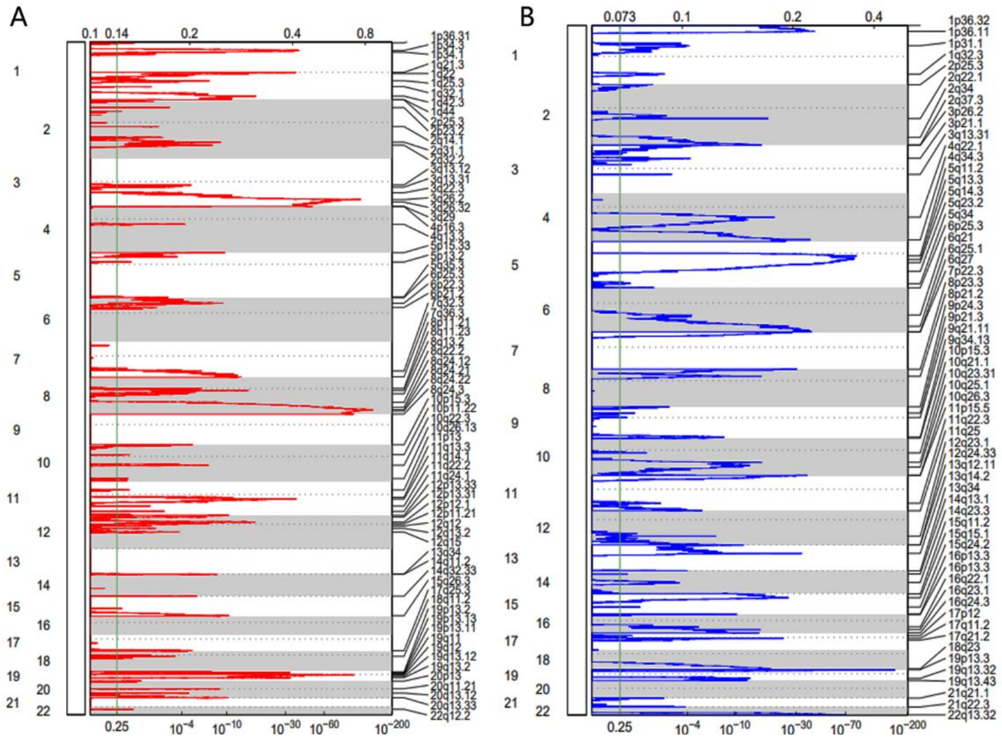

Figure S4 GISTIC analysis revealed the TCGA genome distribution of copy number alterations.

A. GISTIC q-values (x-axis) for deletions (blue) are plotted across the genome (y-axis). B. GISTIC q-values (x-axis) for and amplifications (red) are plotted across the genome (y-axis).

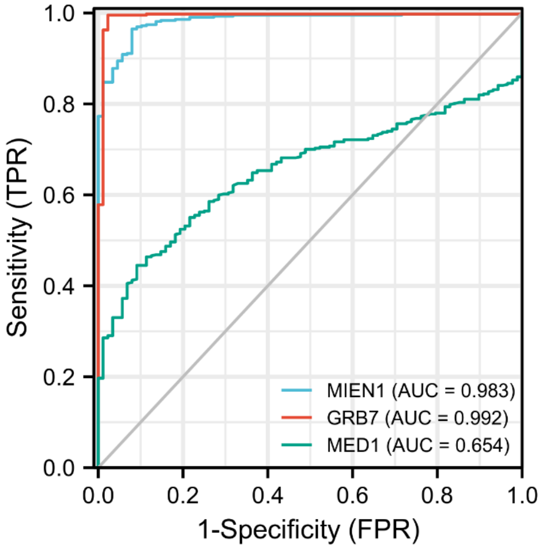

Figure S5 ROC of three driver genes (MIEN1, GRB7 and MED1) shows they are of high diagnostic value in SOC.

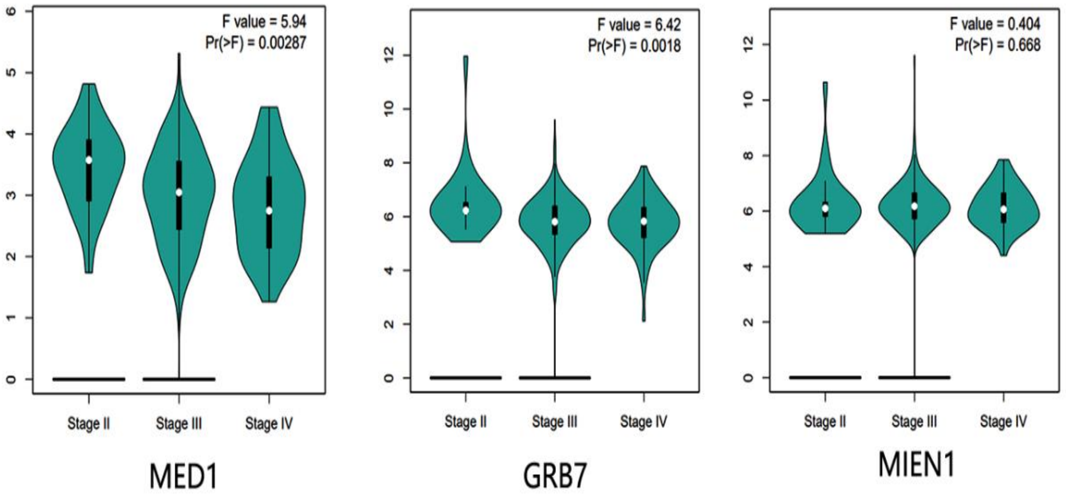

Figure S6 Correlation analysis between three driver genes (MIEN1, GRB7 and MED1) and the pathological stage of SOC.
